# The Treatment of the Acute Inflammations of the Middle Ear

**Published:** 1877-11

**Authors:** 


### Translations.

### THE TREATMENT OF THE ACUTE INFLAMMATIONS OF THE MIDDLE EAR.

By Dr. Weber-Liel.

[Translated from the Deutsche Zeitschrift fur praktische Heilkunde. By Dr. F. C. Hotz.]

The following remarks relate to an otological topic which is of especial interest to practicing physicians. This paper is principally written for the non-specialists.

Many mistakes are made in the treatment of those acute aural diseases which are so often met with, in the practice of every physician. These errors do not always arise from a lack of knowledge of well founded doctrines, but rather from the fact, that the doctrine of the acute aural affections, and especially of the acute inflammations of the middle ear, has not yet been brought into satisfactory shape. The experience of many years of special practice has led me to adopt a different view and treatment, in many points, of the diseases in question, as the teachings of the text-books of acute inflammation did not prove satisfactory to me. I beg to differ with the opinion of those who believe that the acute inflammation of the ear always shows the same character.

They are not always the simple idiopathic affections for which the same remedies have the same effects. Undoubtedly all my professional colleagues have had the sad experience that in some cases the inflammation took a most pernicious course; the earache did not abate, and the hearing power was completely destroyed, although hot water was instilled into the ear with the utmost care, as frequently as recommended; although leeches were repeatedly applied in front and back of the ear, and in spite of cold applications, air douche, fomentation, gargles, and paracentesis of the membrana tympani. And if these remedies were only always applied! But often physicians believe that they have done all in their power by applying a blister behind, or warm poultices upon the ear.

As I said before, my remarks are addressed to those physicians who are not engaged in special aural practice, and are not very familiar with the use of the otoscope. It is my firm belief that many and especially acute cases can be promptly relieved without the use of the otoscope.

Let us first consider those inflammations which so commonly originate from a severe cold, in winter as well as in the warm season. A person who has been afflicted with chronic pharyngitis is taken with a violent cold in the head. During the act of deglutition, he often feels an uncomfortable strain and soreness in the region of the ear, or in the course of the Eustachian tube; he has a sensation of fullness in the ear and a heaviness in the head, and occasionly shooting pains in the ear. These symptoms, and the failure of the Valsavian experiment (the attempt to force air into the middle ear through the Eustachian tube while the mouth and nostrils are closed) clearly indicate that inflammatory irritation has extended into the middle ear. Usually it is confined to one side. Sometimes it happens that the patient trying to blow his nose, feels a sudden, severe, stinging pain in one ear, attended with the sensation as if a foreign substance had got behind the membrana tympani. This uncomfortable sensation continues, and in the subsequent night the patient awakes with earache which does not abate till toward morning. But the ear remains sensitive also during the daytime; the hearing is muffled and there is a roaring noise in the ear. Under favorable circumstances these symptoms gradually subside. With some patients, however, the sensation of fullness, the roaring noise and deafness continue. At night the ear begins to ache again, and the pain will not cease again until a purulent discharge appears in the external meatus.

The membrana tympani, in these cases, has become perforated in consequence of a violent otitis media caused by mucopurulent matter which had accidently been blown from the Eustachian tube into the hyperæmic tympanum. During a severe naso-pharyngeal catarrh, the otitis media has, in many cases, a different origin. In leaving a hot room the patient exposes one ear to a draught of cold air, wind or rain; or he has been sitting in a draught, one side exposed to the open window. At once he experiences an uncomfortable feeling over that side and in that ear. Again it is at night time that the patient is awakened by earache, which does not subside until the discharge of a sero-purulent secretion from the external meatus shows the drum membrane has been perforated. This perforation may take place within a few hours, or after a few days during which time the patient suffers great pain and cannot rest or sleep. The external meatus soon becomes involved in the inflammatory process as is shown by a tumefaction of its texture.

The region in front of the ear is, as a rule, very tender, especially on pressure of the tragus; but the mastoid region becomes sensitive to pressure only, if the affection has lasted some time or been improperly treated. Such cases are simple catarrhal inflammations of the middle ear.

Under a half way proper treatment they will get well within two to six weeks, the at first copious otorhœa gradually decreasing. In a healthy, robust person the loss of substance in the membrana tympani is very limited, the perforation is closed by cicatricial tissue with or without adhesions; but more or less deafness remains, which very seldom disappears spontaneously.

What shall a physician do, residing in the country, unable to procure the services of a specialist, to prevent the otitis from running such a course as to jeopardize the functions of the ear, or even the life of the patient? In the first place, he shall employ the water douche, and then the air bath. For which purpose two instruments are required, which every practitioner ought to possess; viz., the *naso-pharyngeal syringe* and the *air bag*. The latter being for the removal of the secretion from the tympanic cavity, and the syringe for cleansing the naso-pharyngeal space. If the syringe is used previous to the air bath, the latter will be more successful, as it is then sure not to carry any mucus from the orifice of the Eustachian tube into the tympanic cavity. The bulbous nozzle of the syringe charged with a warm solution of common salt or ammonia, is passed into one nostril of the patient, and the syringe emptied by a moderate pressure upon the piston. The lotion is injected into the nasal cavity, whence it passes into the pharyngeal space toward and into the tubal orifices, and after washing away any mucous which may have gathered on these parts, the liquid returns through the other nostril. One must, however, be very cautious in using these injections, for, if too much force be used, or the other nasal cavity be obstructed, the fluid might be forced into the healthy middle ear, causing inflammation. The cleansing of the naso-pharyngeal cavity is followed by the air bath. The bulbous nose-piece of the air bag is firmly inserted into one nostril, the patient takes a little water into his mouth, and swallows it at a given sign (1, 2, 3), while at the same instance the surgeon suddenly compresses the bag. In this manner the air in the naso-pharyngeal cavity is condensed, and will during the act of deglutition escape into the middle ears, provided the Eustachian tubes are not completely obstructed.

This experiment must be repeated several times in succession, after which the patient usually feels greatly relieved. For if the membrana tympani has not been ruptured yet, the tympanic cavity has received a fresh supply of air and been relieved of the abnormal tension and pressure. In young persons, and especially in children, the effect is usually instantaneous; the earache ceases and pressure upon the tragus is not painful, at least, for some time. In some cases, even the morbid process could be aborted, and the suppuration prevented by the repeated employment of the water douche and air bath. Where the membrana tympani has been perforated, the air and water bath serves to cleanse the ear of the purulent secretion, which after this operation is often seen to flow copiously from the external meatus.

A second local remedy in the treatment of catarrhal inflammations of the middle ear, is the application of cold on the ear. Leeches have repeatedly been recommended to be put in front of and behind the ear; but my experience is that they afford temporary relief only, and in many cases do positive harm.

The favorable influence of cold is usually very marked. A small towel is folded, to the size of from six to nine inches square, soaked in cold water, and wrung out well and put on in such a manner that it covers the posterior portion of the face, the ear and the side of the neck. The wet towel is covered with a dry woolen cloth. After half an hour the wet cloth is changed. The change of dressing must be done so quickly as to leave the ear uncovered but a second, as otherwise the patient might catch cold. After a few applications the pain abates. They need now to be changed only every one to two hours, but must be continued for one, two or three days, till the pain does not return. As the pain grows especially severe at night, it is advisable to change the wet dressing oftener after six o'clock in the evening, until the patient sleeps. For simple, uncomplicated cases this local treatment is all that is needed; besides the regulation of the bowels, which always must be attended to.

But in other cases this treatment will not prevent the pain from returning at night and depriving the patient of sleep. Many physicians then, as I have observed, resort to hypodermic injections of morphia, in the region of the ear. No doubt sleep is thereby often procured. But my experience has taught me, that in excitable persons, women particularly, the morphine often produces a morbid nervous irritability, and and gives rise to complaints more troublesome than the original affection; viz., toothache, neuralgia in the ramifications of the fifth nerve, spasm of the masseter and cervical muscles. For internal medication, I can highly recommend the oil of turpentine, to be given in capsules, each containing from 10 to 12 drops; three capsules are taken at noon, and five or six in the evening. The turpentine is continued for two or three days only; nausea and dizziness do not contra-indicate its further use. But if the earache does not subside in the first night, the remedy may as well be discontinued. For children, who are so often seized by earache at night, due to catarrhal inflammation, I prescribe the iodide of potassium. The pain once over, it is only necessary to use the air bath once daily, and to remove all secretions from the external meatus by means of a soft camel's hair brush; the meatus is plugged with cotton and the ear is covered with a cloth. Every two or three days the ear may be syringed with a weak solution of salt.

By this treatment the catarrhal inflammation will completely subside within six to fourteen days, even if the membr. tympani has been perforated and a purulent secretion is discharged from the external meatus. But in order that the hearing power be completely restored, the air douche, with *Gruber's new method*, had better be continued. At the moment when the air bag is suddenly emptied into the nostril, the patient pronounces such syllables as hick, hack or huck, giving particular stress to the enunciation of the k. When, during this experiment, the patient's head, with the affected ear up, is inclined to one side, the air can be distinctly heard to whistle through the perforated membr. tympani. Children need neither swallow water nor enunciate the above syllables, to make the air douche successful; for their Eustachian tubes will be opened by the strain of the pharyngeal muscles, during the act of crying and screaming which is almost inevitable. Should the muco-purulent discharge continue over one week after the painful stage is passed, have the spiritus vini rectificatus may be employed, as I recommended it for the treatment of chronic otorrhœa, years ago. The ear, having been well cleansed, is filled with lukewarm alcohol. If it enters the tympanic cavity it creates an acute pain, which, however, lasts but a minute, and after several applications is not felt at all. By the application of the alcohol the purulent discharge disappears, often most rapidly, and the cicatrization is greatly hastened.

There is a considerable number of acute inflammations of the middle ear, which undoubtedly cannot be accepted as simple catarrhal affections. In these cases the pain is usually very severe, irradiating over the whole head, and depriving the patient of his nightly rest. The exacerbations at night are particularly marked. In the simple catarrhal inflammation the earache subsides as soon as the tension and pressure are relieved by the escape of the pus through the perforated membr. tympani. In the cases under consideration, however, the

4

otorrhœa does not give any relief. It even happens that the patient's attention is not called to the ear as the seat of suffering, until a discharge from the ear is noticed; for the pain extending over the whole head and face, and the sensitiveness of every movement, make the patient believe he is suffering from rheumatism, rather than an aural affection.

The air bath, and all other remedies mentioned above, give but temporary relief, the cold water dressings proving even harmful. The inflammation soon involves the meatus externus. The region in front of the ear as well as around it, becomes tender; the integument over the mastoid process being red and swollen; in fact, all the symptoms indicating, even to an inexperienced observer, that the inflammation has proceeded from the periosteum of the meatus externus to the mastoid process, or has already implicated the mastoid cells. High fever generally accompanies this affection. This inflammation is very dangerous. It attacks almost exclusively adults, especially cachectic individuals and persons who have had syphilis, or have repeatedly been troubled with rheumatism. I have observed cases[1] in which such inflammations of the ear alternated with rheumatic pains in other parts of the body. And I am surprised that other writers have not yet emphasized the rheumatic nature of certain acute aural diseases.

Although the air bath affords but transient relief, it must not be neglected. Cold applications must be avoided. The only local remedy which will relieve the earache is the application of warmth, either in the shape of warm cloths covering the whole side of the head, or as warm poultices; they must be changed often and quickly, in order to prevent the head from becoming cold. It is important to cause a free action of the bowels and the skin. For internal administration I prescribe quinine and salicylic acid.[2]

It is surprising how the most violent earache subsides un-

---

1 See my book on Progressive Schwerhörigkeitsformen.

2 ℞. Quin. muriat.
Ac. salicyl. aa 0, 3.
Sod. bicarbon. 0, 06.
Three or four powders to be taken daily; the last one at 7 P. M.

der this medication, so that the patient is again able to rest at night. In other cases, however, large doses of turpentine seem to operate with better effect. If either treatment proves inefficient, I have found the carbolic acid to be a valuable means to subdue the inflammatory symptoms and the painful exacerbations. Hypodermic injections (one part of carbolic acid to ten parts of water) are made into the most tender spot behind the ear, every morning and evening.

Should all these remedies fail to reduce the inflammation, which is sometimes the case in cachectic persons—should the tumefaction over the mastoid process increase instead of decreasing, the surgeon must not hesitate in making a deep incision down to the periosteum behind the ear parallel with, and half an inch from the insertion of the concha. The incision should be one inch long at least. It is generally followed by a marked relief, even if pus has not been drawn. The warm applications must be renewed. But if the symptoms unmistakably point to an accumulation of pus within the mastoid cells, no time must be lost in treating this dangerous complication as recommended in all works on aural diseases.

I have yet to say a few words regarding the *intermittent otitis*, which has not been described yet in the text-books, because they consider all otitides under the head of catarrhal inflammations. Since I first described the otitis intermittens[1], I have observed a great many cases of acute aural inflammations which belonged to this class, and were treated as intermittent otitis, so speedily cured that I have no hesitation in saying that a great number of acute otitides, particularly of those occurring in spring and fall, are caused by malarial influences. This cause cannnot always, though it may be often traced. The reason that this form of otitis has hitherto escaped the notice of observers, is I think, that almost every acute otitis shows a pronounced remittent character; during the day the fever and pain subside; at night they increase in violence to abate again toward morning.

The following symptoms are characteristic:

---

1. Monatschrift f. Ohrenheilkunde No. 11, 1871, and Deutsche Klinik, No. 5, 1874.

The disease is ushered in with a more or less pronounced chill, attended by an uncomfortable sensation and roaring noise in the ear. The next day a violent angina and naso-pharyngeal catarrh make their appearance, but on the second day, or night, the patient has another chill, followed by increased roaring and by a severe pain, mostly in one ear. The earache lasts until morning. After a few hours' sleep the patient awakes, and is bathed in perspiration. During the day he feels tolerably well, only having an uncomfortable feeling in the ear, but being more annoyed by the symptoms of the pharyngitis. But the more these latter symptoms subside the more violent the noise and pain in the ear become. Again, they return in the evening, or at night, preceded by a chill, and in the third or fourth night pus is copiously discharged from the ear. Not till then the patient calls upon his physician, because he had been deceived about the nature of the disease by the daily respite from pain. The examination generally reveals the signs of the simple, so-called catarrhal otitis; sometime a perforation of the membrana tympani with a purulent discharge. Pressure upon the tragus, which in all other aural inflammations is also very tender during the day, is not painful during the intermissions, at least in the beginning of the disease; nor is there any fever.

The physician employs the remedies usually recommended as effective for catarrhal affections, or those I have mentioned above; nevertheless he will see the symptoms aggravated in the following night. If there is no otorrhœa, and the physician detects an accumulation of pus behind the membran. tymp., which he thinks causes the increased pain, he will find by experience, that the paracentesis of the membrane and the evacuation of the pus are of no benefit. Very soon the mastoid process is implicated, and the free intermissions become less marked when the mastoid cells are filled with pus.

No air bath, cold or warm applications, injections of morphine, nothing in short, can prevent the return of the fearful exacerbations at night. Even if the mastoid cells have been opened, and a free outlet for the pus has been established, the painful symptoms will continue always to become exacerbated at a certain time. The Eustachian tube is in the most cases scarcely pervious to inflation, even if there is no naso-pharyngeal catarrh; the walls of the tube are relaxed. In all such cases we must ascertain whether the patient has chills at certain hours, and at regular intervals we must use the thermometer, inquire as to profuse perspirations, and examine the spleen, which very often is quite sensitive to pressure.

The therapy must consist in *large doses of quinine.* I generally prescribe,

| | | |
|---|---|---|
| ℞. | Quin. muriat, - - - | 0.5 to 1.0 grammes. |
| | Morph. muriat, - - | 0.02 |
| | Rhei. pulv. - - - | 0.06. M. |

At noon the patient takes ½ powder, and one powder in the evening, about 1½ hours before the return of the chill and pain. The quick and decided effect of this treatment is often surprising.

The effect is less determined in neglected cases, because of complications then already created which require the local treatment mentioned above; I have found that in protracted cases of this intermittent inflammation the perforation of the mastoid process is often urgently indicated in order to establish a free outlet for the pus, which, under an improper treatment, accumulated in the mastoid cells. But the morbid process itself is effectually arrested by the administration of quinine.

